# Antibacterial and antibiofilm activity of silver nanoparticles stabilized with C-phycocyanin against drug-resistant *Pseudomonas aeruginosa* and *Staphylococcus aureus*

**DOI:** 10.3389/fbioe.2024.1455385

**Published:** 2024-10-23

**Authors:** Zahra Chegini, Aref Shariati, Mohammad Yousef Alikhani, Maliheh Safaiee, Shahin Rajaeih, Mohammadreza Arabestani, Mehdi Azizi

**Affiliations:** ^1^ Department of Microbiology, School of Medicine, Hamadan University of Medical Sciences, Hamadan, Iran; ^2^ Infectious Diseases Research Center (IDRC), Arak University of medical sciences, Arak, Iran; ^3^ Department of Organic Chemistry, Faculty of Chemistry and Petroleum Sciences, Bu-Ali Sina University, Hamedan, Iran; ^4^ ENT and Head and Neck Research Center and Department, The Five Senses Health Institute, Iran University of Medical Sciences, Tehran, Iran; ^5^ Infectious Diseases Research Centre, Hamadan University of Medical Sciences, Hamadan, Iran; ^6^ Department of Tissue Engineering and Regenerative Medicine, Hamadan University of Medical Sciences, Hamadan, Iran

**Keywords:** silver nanoparticles, C-phycocyanin, biofilms, antibiotics-resistant, new treatment

## Abstract

**Background:**

Biofilms are bacterial communities that can protect them against external factors, including antibiotics. In this study, silver nanoparticles (AgNPs) were formed by modifying AgNPs with C-phycocyanin (Ag-Pc) to inhibit the growth of carbapenem-resistant *Pseudomonas aeruginosa* (CR *P. aeruginosa*) and methicillin-resistant *Staphylococcus aureus* (MRSA) and destroy biofilm of these bacteria.

**Methods:**

The AgNPs were prepared with the green synthesis method, and Pc was used to stabilize the AgNPs. The Ag-Pc’s antibacterial and antibiofilm effects were evaluated using the Microbroth dilution method and microtiter plate assay. The inhibitory effect of Ag-Pc on the expression of biofilm-related genes was evaluated by real-time PCR. Moreover, the MTT assay was used to assess the Ag-Pc toxicity.

**Results:**

The Ag-Pc minimum inhibitory concentration (MIC) was 7.4 μg/mL for CR *P. aeruginosa* and MRSA. Pc did not show antibacterial effects against any of the strains. Ag-Pc suppressed biofilm formation and destroyed matured biofilm in both bacteria more efficiently than the AgNPs (P< 0.05). The expression of all genes was not significantly reduced in the presence of synthesized nanoparticles. Finally, the MTT assay results did not show toxicity against a murine fibroblast cell line (L929) at MIC concentration.

**Conclusion:**

The present study showed the promising potential of Pc for improving the antibacterial and antibiofilm function of AgNPs and inhibiting drug-resistant bacteria. Therefore, Ag-Pc nanoparticles can be considered a promising therapeutic approach for the managing of the bacterial biofilm.

## 1 Introduction

Over 2.8 million individuals in the United States are affected annually by multi-drug resistant (MDR) bacterial infections. It is estimated that by 2050, the fatality rates of these illnesses will exceed those of cancer (Park et al., 2023). The problem posed by MDR bacteria is exacerbated by their capacity to develop biofilm infections (Vestby et al., 2020). The compact arrangement of the extracellular polymeric substances (EPS) secreted by bacteria can impede the infiltration of immune cells from the host and antibiotics, enabling bacteria to survive for extended periods (Bjarnsholt, 2013). According to reports, biofilm-associated infections comprise over 80% of recurring and long-lasting microbial infections (Park et al., 2023).

*Pseudomonas aeruginosa* and *Staphylococcus aureus* are significant human pathogens that cause wound and skin disorders, food poisoning, and nosocomial infections (Bhowmick and Koul, 2016; Bahramian et al., 2019). Conventional antibiotics and antimicrobials face difficulties in treating the biofilm community of these bacteria. Consequently, in recent years, there has been a focus on finding novel approaches to address treatment failures due to the rise in antibiotic resistance. Nanoparticles are currently extensively employed in biomedicine, cosmetics industry, and environmental management due to their distinctive physical and chemical characteristics and potent bactericidal impact (Guo et al., 2019; Shariati et al., 2022; Vasiliev et al., 2023).

Metal nanomaterials, such as silver nanoparticles (AgNPs), can emit metal ions that can deactivate microorganisms (Fan et al., 2018). Upon touching bacteria, AgNPs adhere to the surface of the bacterial cell wall and undergo oxidation. This process releases concentrated Ag + ions at the interface between the nanoparticles and the bacterial cells (Vasiliev et al., 2023). To this end, silver ions (Ag+) can induce oxidative stress and damage DNA in bacterial cells (Namivandi-Zangeneh et al., 2021). Nevertheless, the excessive release of metal ions can lead to toxicity in the body and cause harm to local tissues as a side effect at large doses (Krishnan et al., 2020). Additionally, single AgNPs form aggregates because of their tiny size and high surface potential. (Dutra-Correa et al., 2018). AgNPs are prone to oxidation in real-world applications, decreasing their antibacterial effectiveness. To resolve this issue, many techniques have been employed to alter the properties of AgNPs (Enan et al., 2021). There is a growing trend to create environmentally friendly methods for producing AgNPs used in clinical and biomedical settings (Wei et al., 2018). The production of AgNPs using biomolecules results in AgNPs that are highly soluble in water and have excellent biocompatibility. Additionally, these AgNPs can be easily modified or tailored for specific applications. Hence, proteins, peptides, DNA, and other compounds can be highly effective templates for creating AgNPs (Thangam et al., 2013).

AgNPs can be synthesized using several techniques. The production of AgNPs using conventional techniques, such as chemical reduction and physical procedures, is frequently linked to environmental and health issues. These traditional techniques for producing AgNPs require chemical reagents and potent reducing agents, which give rise to substantial environmental and health problems because of the production of dangerous by-products and the depletion of finite resources (Dubey et al., 2023). Within this particular framework, the advancement of green synthesis methods for AgNPs has become increasingly important as a non-toxic and recyclable alternative (Muthuraman et al., 2019). Green synthesis techniques employ natural sources, including plant extracts, microbes, and biopolymers, as active agents for decreasing and stabilizing AgNPs throughout their production (Dubey et al., 2023). These techniques have numerous benefits compared to traditional chemical procedures, including ecologically sustainable. Noteworthy, green synthesis methods aim to significantly reduce the environmental impact of nanoparticle manufacturing by minimizing the use of hazardous chemicals, organic solvents, and energy-intensive procedures. Green synthesized AgNPs have improved biocompatibility and decreased cytotoxicity compared to chemically synthesized equivalents, rendering them appropriate for biomedical uses, including drug delivery, imaging, and tissue engineering (Le Ouay and Stellacci, 2015). The availability, renewability, and cost-effectiveness of natural sources for green synthesis allow for the scalable yield of AgNPs with minimal capital expenditure. Green synthesis techniques provide meticulous manipulation of reaction parameters to achieve fine control over the size, shape, and surface chemistry of AgNPs, therefore allowing for customization to specific applications (Hussain, 2022). Collectively, green-synthesized AgNPs are well acknowledged for their biomedical and pharmacological uses. They are environmentally benign, economically efficient, readily expandable, and yield higher quantities than chemically manufactured AgNPs (Habeeb Rahuman et al., 2022).

Phycocyanin (Pc) is a crucial pigment protein in cyanobacteria that significantly functions in photosynthesis (Wei et al., 2018). The phycocyanin complex has three main components: C-phycocyanin (C-Pc), allo-phycocyanin, and R-phycocyanin (R-Pc). C-phycocyanin is frequently employed as a natural pigment in food and cosmetics as a substitute for artificial dyes. Due to their unique characteristics, C-Pcs have found applications in various immunological assays and as fluorescent markers for cell sorting. Moreover, C-Pc and other phycobiliproteins have a high molar absorptivity at visible wavelengths. This characteristic makes them well-suited for use as markers in isoelectric focusing, gel electrophoresis, and gel exclusion chromatography. It has been noted that C-Pc can impede cell growth, affect mammalian cell lines’ gene regulation, and cause malignant cell lines to apoptosis (Liu et al., 2000; Cherng et al., 2007). The protein C-Pc contains functional groups, including amino, carboxyl, and mercapto, which can be protective agents for nanoparticles when creating AgNPs. The silver atom has a pronounced attraction towards these functional groups, resulting in the stable synthesis of nanomaterials (Guo and Irudayaraj, 2011).

Therefore, this study attempts to further improve AgNPs antibacterial function by using Pc for stabilization and investigating these nanoparticles’ antibacterial and antibiofilm function against carbapenem-resistant *P. aeruginosa (*CR *P. aeruginosa*) and methicillin-resistant S*. aureus* (MRSA).

## 2 Materials and methods

### 2.1 Green synthesis of AgNPs

To perform green synthesis of AgNPs, 0.0197 gr of Pc was introduced into a boiling solution of AgNO_3_ (2 mM) with a volume of 20 mL. The boiling process was maintained for 10 min. Subsequently, the solution was left undisturbed at room temperature until the colorless solution transitioned to a yellowish tint, indicating the creation of AgNPs. In the chemical synthesis process, a solution of AgNO_3_ (2 mM) is heated to boiling, and then trisodium citrate is gradually introduced into the boiling solution while stirring. After a little period, the colorless solution became pale yellow.

### 2.2 Characterization of synthesized nanoparticles

The reduction of Ag ions was measured using a UV-Vis spectrophotometer (SPECTOR 250, Analytic Jena) within the wavelength range of 250–800 nm. The solution of generated nanoparticles was diluted, and the amount of Ag ions was measured using inductively coupled plasma-optical emission spectrometer (ICP-OES) Spectroscopy (Perkin-Elmer, 5300 DV). Structural properties have been found using X-ray diffraction (XRD, Cu Kα radiation, Rigaku D/max 2550 V, Japan) analysis. The shape and size of the nanoparticles were determined using Transmission Electron Microscopy (TEM) with a Zeiss Leo q06 instrument running at an accelerating voltage of 200 kV. To do this, 10 μL of aliquots of nanoparticle solution were drop-casted onto a carbon-coated copper grid. The grid was then placed on paper to remove any excess solvent. The ELSZ-1,000 zeta-potential and particle sizer (Mastersizer 2000, Malvern, United States) were utilized to determine the average particle size, distribution, and stability of the AgNPs. The powdered AgNPs were analyzed using FTIR spectroscopy (Tensor 27, Bruker Co.) with a frequency range of 4,000–400 cm^-1^ and a resolution of 4 cm^-1^. The KBr pellet method was employed for the analysis.

### 2.3 Hemolysis assay

A 2 mL of freshly anticoagulated blood was combined with 2.5 mL of PBS to conduct the hemocompatibility test. The diluted blood, adjusted to a volume of 150 μL, was subjected to the samples for 1 h at a temperature of 37°C. Negative and positive controls were generated by diluting blood with PBS (without lysis) and deionized water (with a lysis level of 100%), respectively. After incubation, the samples were centrifuged at 1,500 revolutions per minute for 5 min to yield plasma. To evaluate the hemolytic capacity of the Ag-Pc, the absorption of hemoglobin released into plasma was quantified at a wavelength of 545 nm using a Microplate Reader. Three replications of the experiment were conducted for each test solution (Liu et al., 2014).

### 2.4 Cytotoxicity study (MTT assay)

The cytotoxicity assay test was performed using the MTT assay kit from Kiazist, Iran. The cytotoxic effects of Ag-Pc were evaluated using a murine fibroblast cell line (L929). The kit contained a 96-well clean plate, two-channel reservoirs, solvent, and MTT reagent. 10^4^ L929 fibroblasts were enumerated using the trypan blue staining technique in this stage. Subsequently, these cells were transferred to 96-well cell culture plates filled with Dulbecco’s Modified Eagle’s Medium (DMEM) culture supplemented with 10% fetal bovine serum (FBS) and 1% penicillin-streptomycin. The cells were then incubated at a temperature of 37°C with 5% CO_2_ for an overnight duration. After removing the DMEM medium, Ag-Pc was introduced with DMEM, which contained 10% FBS and was incubated for 24 h. The positive controls consisted of wells containing medium without any medication. Each experiment was conducted three times. After performing a PBS wash to eliminate residual drugs or polymers, each well was filled with 150 µL of fresh DMEM without fetal bovine serum. Subsequently, 20 µL of the MTT test reagent was added to each well. The plate was placed in an environment with a temperature of 37°C and a carbon dioxide concentration of 5% for 3–4 h. Subsequently, a 100 µL solubilizer was added to each well, followed by agitation on an orbital shaker for 15 min to disperse formazan particles. A 96-well ELISA plate reader was utilized to quantify the absorbance at a wavelength of 570 nm. The vitality of the cells was determined by calculating the absorption percentage of the cells in the positive control group, where all cells were alive (100 percent alive) (Nouruzi et al., 2023).

### 2.5 Bacterial strains

The clinical isolates of MRSA and CR *P. aeruginosa* were obtained from wound infections at *Besat* Hospital in Hamadan, Iran. Subsequently, the bacteria were moved to a new growth medium and placed in an incubator at 37°C until they reached the mid-logarithmic phase. The control strains utilized in this investigation were obtained from Hamadan University of Medical Science in Hamadan, Iran.

### 2.6 Bacteria growth inhibition by microbroth dilution assay

The broth microdilution method, following the directions of the Clinical & Laboratory Standards Institute (CLSI), was used to determine the minimum inhibitory concentration (MIC) and minimum bactericidal concentration (MBC) (Lewis and James, 2023). Bacterial isolates were cultured overnight under suitable circumstances and in the appropriate medium. The initial stock solution was diluted in steps ranging from 956.70 to 3.7 μg/mL. Subsequently, serial dilutions of Ag-Pc and Pc, starting at a concentration of 956.70 μg/mL, were added to 96-well plates. Each well was inoculated with 10 μL of bacteria in Mueller-Hinton broth (MHB), with a final concentration of 5 × 10^5^ CFU/mL. The MIC was established as the lowest concentration of Ag-Pc and Pc, and it did not exhibit any apparent growth after 24 h of incubation at 37°C. In addition, the MBCs were determined by evaluating the lowest dilution that resulted in no growth (>99%) after overnight incubation on Mueller-Hinton agar (MHA). The experiments were conducted in triplicate, and *S. aureus* ATCC 25923 and *P. aeruginosa* ATCC 27853, and un-inoculated MHB media were considered positive and negative controls, respectively (Mirzaei et al., 2022b).

### 2.7 Biofilm formation assay

CR *P. aeruginosa* and MRSA’s capacity to generate biofilms was evaluated using a microtiter plate (MTP) assay. Initially, the isolates were introduced into a 2 mL TSB medium that included 1% glucose (1% Glu TSB). The mixture was then placed in an incubator and kept at 37°C for 24 h. The following day, a 0.5 McFarland turbidity was created by measuring the absorbance of the bacterial suspension at a wavelength of 625 nm. Subsequently, 100 μL of the bacterial suspension, containing 10^7^ colony-forming units (CFUs), was combined with 900 μL of 1% Glu TSB in a sterile tube. Then, 200 μL of this produced suspension, containing 2 × 10^6^ CFUs, was applied to each well of 96-well polystyrene microplates. The microplates were incubated at 37°C for 24 h at 75 rpm. Subsequently, the contents of the wells were carefully emptied, and the wells were rinsed three times with normal saline and subsequently dried with air. Afterward, 200 μL of pure methanol was added to each well to immobilize the formed biofilm. After 15 min, the liquid in the wells was removed, and the wells were again allowed to dry in the air. Next, the wells were treated with 200 μL of a 0.05% crystal violet solution for 5 min. The solution was then removed, and the wells were rinsed three times with normal saline and let to dry in the air once more. After that, 200 μL of 100% ethanol was added to each well and agitated for 30 min at 37°C. Finally, each well’s optical densities (ODs) were measured at a wavelength of 600 nm. The ability of the studied bacteria *S. aureus* ATCC 25923 and *P. aeruginosa* ATCC 27853 to form biofilms was classified as follows: non-biofilm forming (OD_test_ ≤ OD_control_), weak biofilm forming (OD_control_ < OD_test_ ≤ 2 × OD_control_), moderate biofilm-forming (2 × OD_control_ < OD_test_ ≤ 4 × OD_control_), and strong biofilm-forming (4 × OD_control_ < OD_test_) was considered as three standard deviations (SDs) above the mean OD of the negative control. The biofilm development test was replicated thrice (Mirzaei et al., 2022b).

### 2.8 Minimal biofilm inhibitory and eradication concentration (MBIC and MBEC)

As previously stated, a microbroth dilution test was utilized to ascertain the sub-MIC concentrations. A 100 μL volume of a diluted bacterial suspension containing 10^6^ CFU/mL, mixed with TSB medium, was added to each sterile 96-well microtiter plate well. After introducing various concentrations of Ag-Pc into each well, the microplate was left to incubate at 37°C for a night. Following overnight incubation, the contents of the wells were carefully removed and cleaned three times with a standard saline solution. Ultimately, the amount of biofilm created was determined using the microtiter plate test, as previously described. Every experiment was conducted three times for each strain. The optical densities of the wells were measured at a wavelength of 600 nm. The MBICs were calculated as follows (Bardbari et al., 2018; Mirzaei et al., 2022a):

$$\text{Biofilm inhibition }\%=\left[1-\left(\text{OD test}/\text{OD control}\right)\right]\times 100$$

In an alternative approach, bacteria are initially let to develop biofilm, followed by an assessment of the effectiveness of Ag-Pc and Pc in combating these biofilms. Initially, the isolates were introduced into a 2 mL TSB medium that contained 1% glucose. Subsequently, they were placed in an incubator at 37°C for 24 h. Afterward, 200 µL of a diluted bacterial solution (1 × 10^6^ CFU/mL) was introduced into a polystyrene 96-well microtiter plate. The plate was then incubated for 24 h at a temperature of 37°C. After the incubation period, the contents of each well were discarded, and the wells were washed three times with 200 μL of sterile PBS (pH 7.4). In the experiment, 100 μL of Ag-Pc were applied to each well. The samples were then incubated at 37°C for 24 h. After emptying the wells, they were rinsed three times with PBS. Then, 100 μL of a new saline solution was added to the wells. After scratching and mixing, 10 μL of the well contents were cultured on MHA at 37°C for 48 h, and the resulting colonies were counted. The MBEC values for Ag-Pc and Pc were determined as the minimum quantity of antibiotics necessary to eradicate 99.9% of the bacteria within the embedded structure (Mirzaei et al., 2022a).

### 2.9 Assessing the impact of Ag-Pc on the expression of biofilm-related genes

The assessment of gene expression associated with biofilm development was conducted using RT-PCR. MRSA and CR *P. aeruginosa* were grown in TSB with a sub-MIC of Ag-Pc at 37°C for 12 h. The mature biofilms were formed, and then the total RNA was extracted using the RNX-Plus solution, following the procedure provided by the manufacturer (Sinaclon, Iran). Subsequently, RNA quality and concentration were assessed by measuring the OD using a Nano-Drop spectrophotometer. An RNA-primer mixture containing 1 µL of Oligod (T) primer, 1 µL dNTPs mix, and 7 µL DEPC-treated water was prepared for cDNA synthesis. This mixture was added to 1 µL of extracted RNA and incubated at 70°C for 5 min. Then, the cDNA synthesis mixture was prepared with 4 μL 5X Buffer M-MuLV, 1μ M-MuLV Reverse Transcriptase, 0.5 μL RNase inhibitor and 4.5 μL DEPC-treated water. 10μL of the cDNA was added to each RNA-primer mixture and incubated at 50°C for 30–50 min and 70°C for 15 min. Chill the tubes on ice and collect the solution by centrifuging the tube briefly. The cDNA was stored at −20°C. The SAYBER GREEN Master Mix (Amplicon Without ROX) was used in this experiment. The reaction mixture consisted of 2 μL of cDNA and 1 μL of each primer specific to the *icaA*, *lasR*, and *algD* genes, as well as the 16s rRNA gene. The total volume of the reaction mixture was 20 μL. Noteworthy, the LightCycler^®^ (LightCycler^®^ 96 Instrument, Roche, United States) instrument was used in the current study, and the primer sequences utilized in the experiment are documented in Table 1. The thermocycling methodology involved an initial preheating stage at 95°C for 10 min, followed by 40 cycles of denaturation at 95°C for 15 s, annealing at 60°C for 45 s, and extension at 72°C for 30 s. After completing the PCR cycling procedure, data on the melting points were gathered, and a dissociation curve was analyzed for each well. Furthermore, the gene expression was assessed utilizing the ΔΔCt technique (Mirzaei et al., 2022a).

**TABLE 1:** List of specific primers used in this study.

Gene	Sequence	Ref
*Ica*-F *Ica*-R	AAAGATGTAGGTTATTGGGATACTGACA CATAGAGCACGTGGTTCGTACTTAA	Vandecasteele et al. (2003)
*lasR*- F *lasR*-R	AAGTGGAAAATTGGAGTGGAG GTAGTTGCCGACGACGATGAAG	Hassan and Abu-resha (2022)
*algD*-F *algD*-R	CTA​CCA​GCA​GAT​GCC​CTC​GGC ATGCGAATCAGCATCTTTGGT	Sonbol et al. (2015)
16s rRNA-F 16s rRNA-R	ACTTCGGGAAACCGGAGC ACCGTGTCTCAGTTCCAG	Heydari et al. (2019)

### 2.10 Statistical analysis

The ANOVA test was employed to assess the disparities among the treatments. The Dunnett test was another statistical method used to compare different groups. Significant differences were seen between the two when the P-value was less than 0.05.

## 3 Results

### 3.1 Characterization of Ag-Pc

**FIGURE 1:**
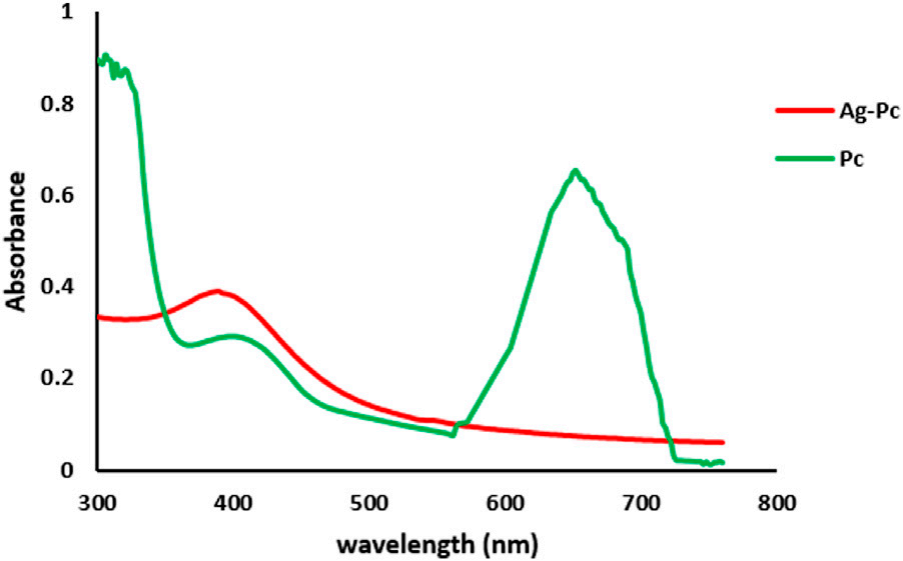
UV-vis spectra of Pc and Ag-Pc were produced using green methods.

The UV-Vis spectrum was utilized to determine the diminished concentration of AgNPs in the solution. The current study utilized UV-Vis analysis to determine the maximal absorption peaks of Pc and Ag-Pc, found at 643 and 398.5 nm, respectively (Figure 1). The presence of those peaks suggests that the Ag-Pc was synthesized. Visual and laboratory analysis showed that synthesized nanoparticles were stable after 3 months (Supplementary Figure S1).

**FIGURE 2:**
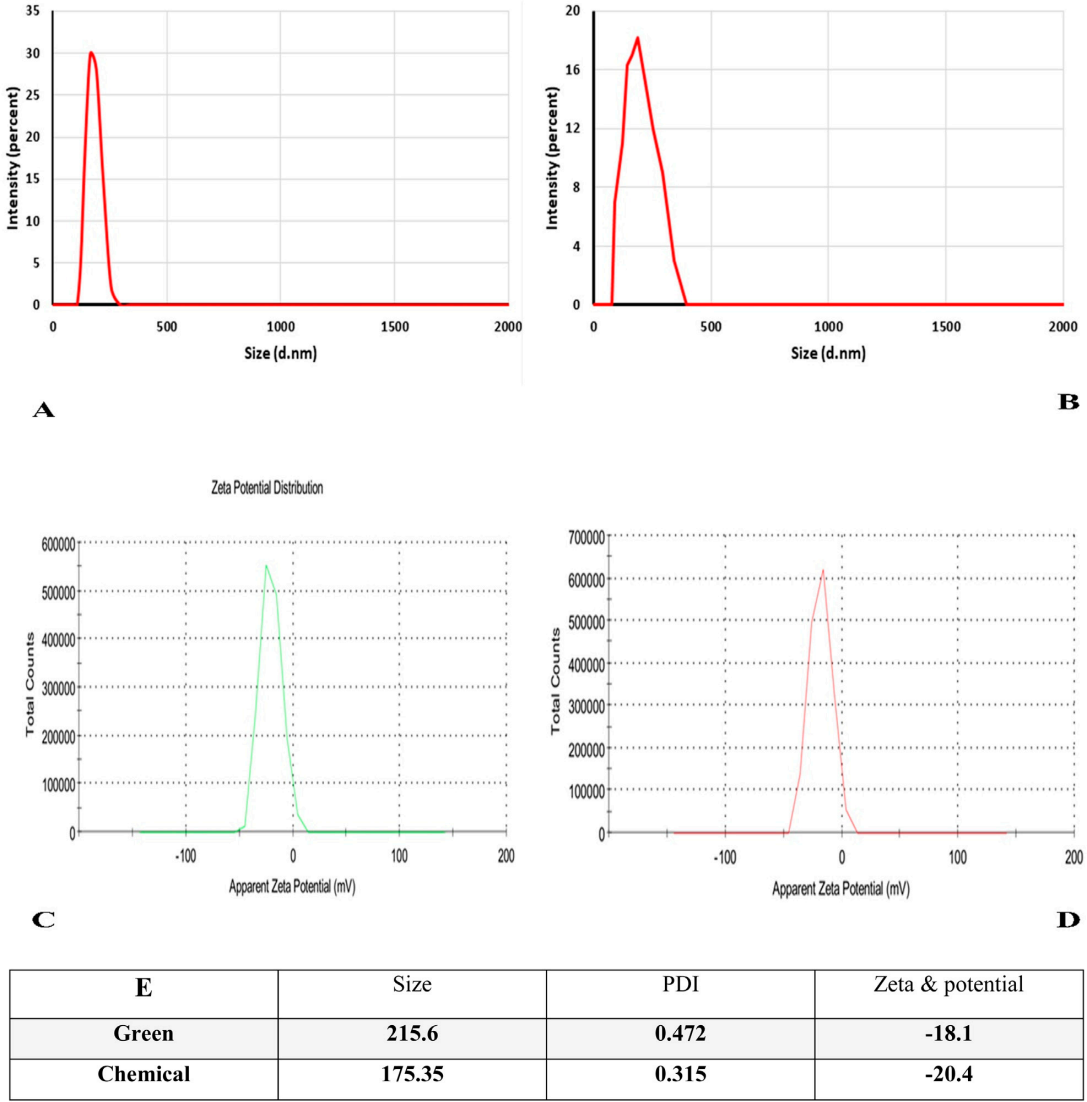
Analysis of DLS profile and zeta potential of nanoparticles made using green and chemical methods. **(A)** the Size Ag-Pc, **(B)** the size of chemically synthesized AgNPs. **(C)** and **(D)** zeta potential of green and chemical synthesized Ag-Pc, respectively. **(E)** Table shows synthesized nanoparticles’ polydispersity index (PDI), size, and zeta potential.

The hydrodynamic size of the generated nanoparticles was determined using the DLS method. The average particle size of the green synthesized Ag-Pc nanoparticles was 215.6 nm, whereas the chemically synthesized AgNPs had an average particle size of 175.25 nm. The Zeta potential of the Ag-Pc synthesized using green methods was −18.1. This result confirms that the AgNPs have a high level of stability. Noteworthy, the Zeta potential of chemically produced AgNPs was −20.4 mV, as shown in (Figure 2).

**FIGURE 3:**
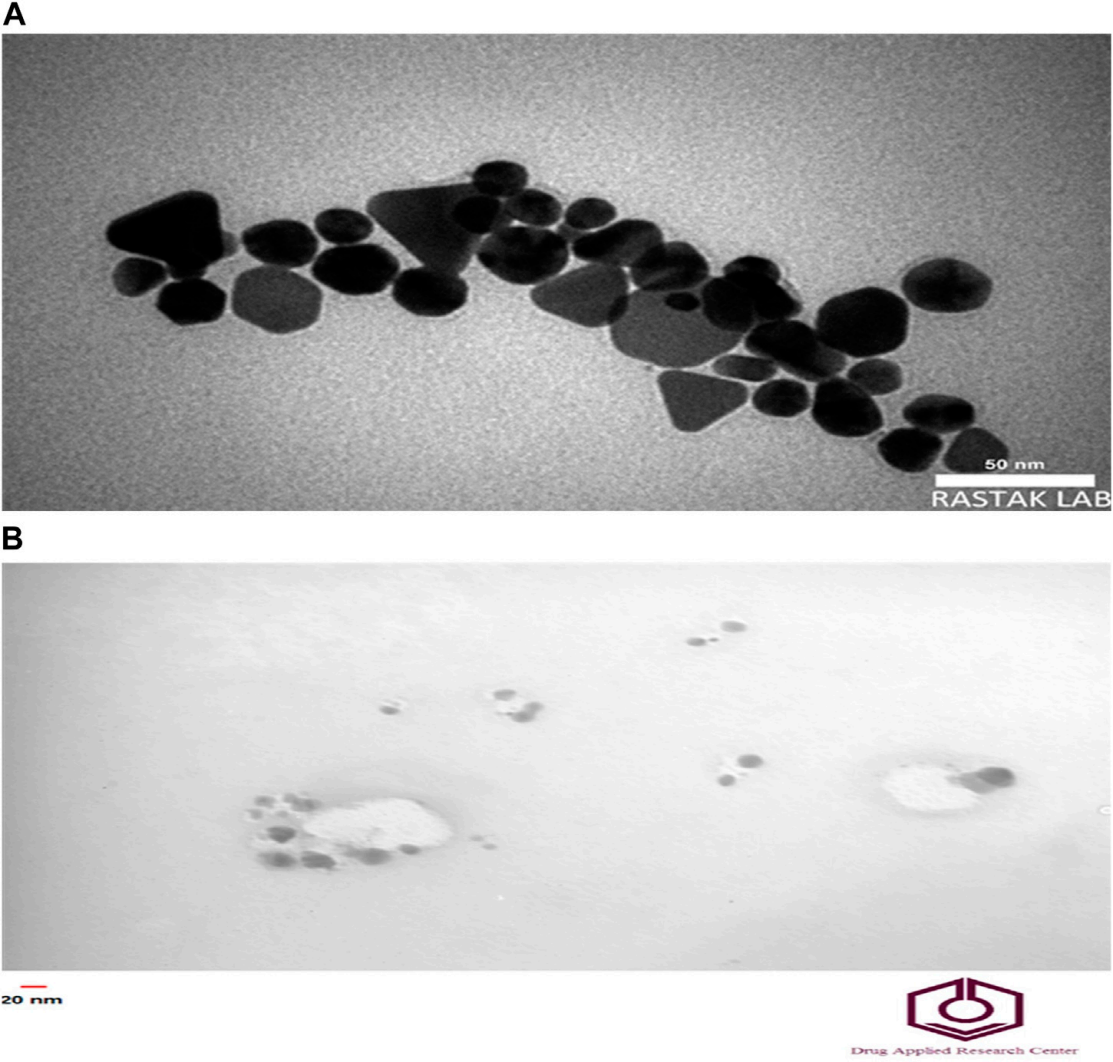
TEM image of nanoparticles. TEM imaging of **(A)** Ag-Pc and **(B)** chemically synthesized AgNPs.

The morphology and sizes of AgNPs were analyzed using the TEM technique (Figure 3). The TEM images revealed that the nanoparticles were dispersed separately, exhibiting shapes like hexagons and spheres. The size of the Ag-Pc NPs ranged from 26 to 43 nm. The TEM investigation revealed that chemically produced AgNPs exhibited a size range of 15–25 nm.

**FIGURE 4:**
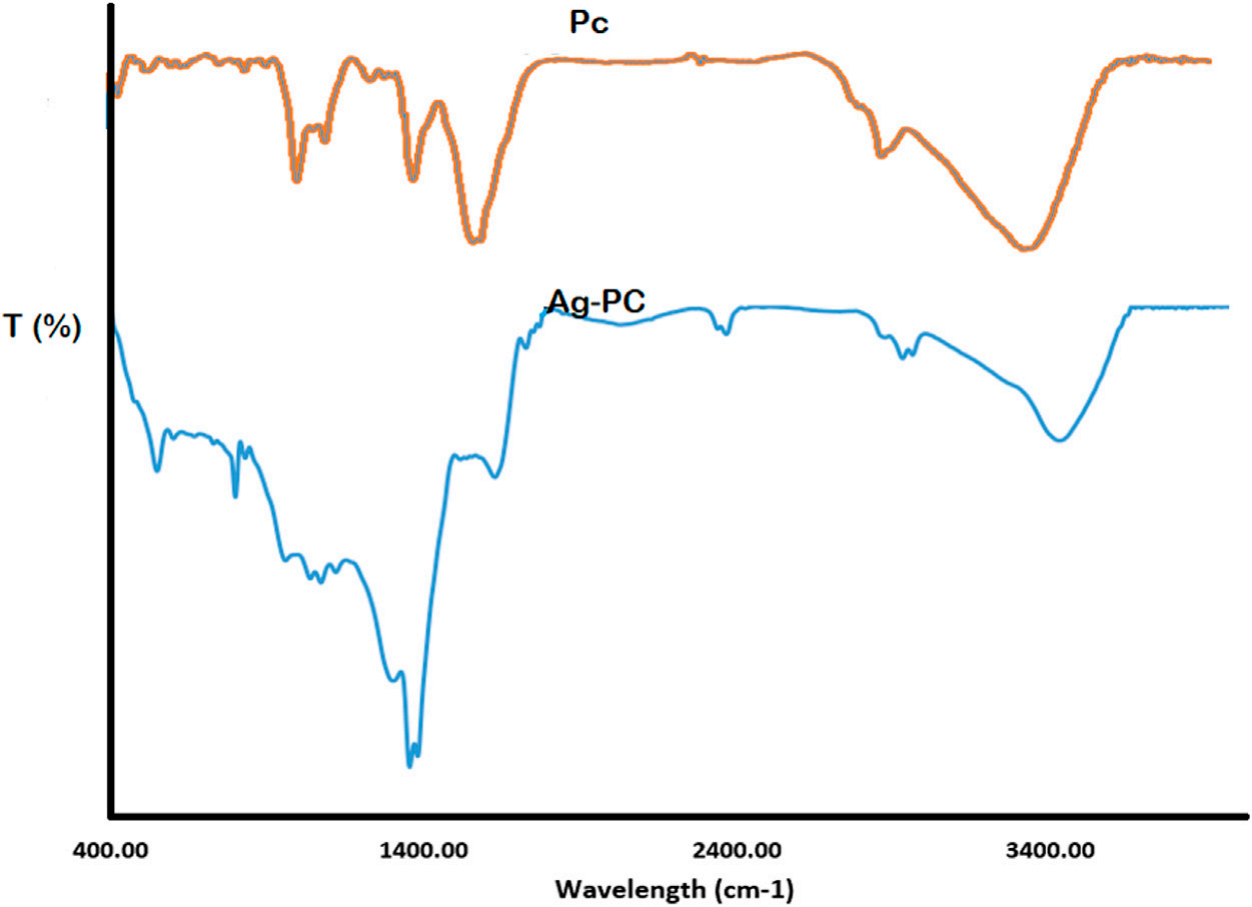
The FTIR spectra of Pc and capped reducing phytoconstituents responsible for forming AgNPs.

The FTIR spectrum of Pc peaks at 3,441 cm^-1^, attributed to O–H bonds stretching in phenolic compounds, water, and fatty acids (Figure 4). The band at 2,933 cm^-1^ corresponds to the stretching of the C–H bonds in the methylene group found in esters, fatty acids, and aliphatic hydrocarbons. The band at 1738 cm^-1^ corresponds to stretching the C = O bonds in ketones, esters, aldehydes and fatty acids. The band at 1,620 cm^-1^ corresponds to the bending of water’s (H–O–H) bonds. The band at 1,517 cm^-1^ corresponds to stretching the C = C bonds in aromatic compounds. The band at 1,462 cm^-1^ corresponds to the in-plane bending of the C–O–H bonds in fatty acids and other compounds. The band at 1,269 cm^-1^ corresponds to stretching the C–O bonds in esters and fatty acids. Lastly, the band at 1,044 cm^-1^ corresponds to stretching the C–O bonds in alcohols and phenols.

Upon comparing the infrared spectra of Pc and Ag-Pc, it is evident that the peak at 3,435 cm^-1^ is present in both compounds. Notably, the peaks at 2,928 cm^-1^, 1,635 cm^-1^, 2,460 cm^-1^, 1,508 cm^-1^, and 1,127 cm^-1^ are present in Pc and Ag-Pc. Compared to the AgNPs and Pc spectrum, these peaks have diminished or risen and shifted to higher or lower wavenumbers. The band observed at 3,435 cm^-1^ corresponds to the stretching of O–H bonds. The band at 2,928 cm^-1^ is attributed to the stretching of methyl C–H bonds in esters. The band at 1,635 cm^-1^ is assigned to bending H–O–H bonds. The band at 1,383 cm^-1^ is attributed to the bending of symmetrical methyl C–H bonds in esters. Finally, the band at 1,127 cm^-1^ is assigned to stretching C–O bonds in carbohydrates and esters. The peak at 2,928 cm^-1^, associated with stretching C-H bonds in aliphatic fatty acids, esters, and hydrocarbons in the Pc, decreased in intensity and migrated to 2,923 cm^-1^. The intensity of the band at 1,635 cm^-1^, which is associated with the bending of water molecules (H-O-H), decreased and shifted to the same frequency of 1,635 cm^-1^. The band at 1,508 cm^-1^, associated with stretching the C = O bond in esters, aldehydes, ketones, and fatty acids, was no longer present.

Additionally, the peak at 1,383 cm^-1^, attributed to the methyl symmetrical C–H bending of esters, exhibited a distinct sharpness. The presence of peaks in the spectrum indicated that the nanoparticles were coated with Pc, which contained several functional groups like carboxylic acid, ketone, aldehyde, and others. The presence of these functional groups results from the nanoparticles’ stability. The statement affirms that phytoconstituents stabilize nanoparticles produced from the Pc via functional groups (Song et al., 2009; Bagherzade et al., 2017).

**FIGURE 5:**
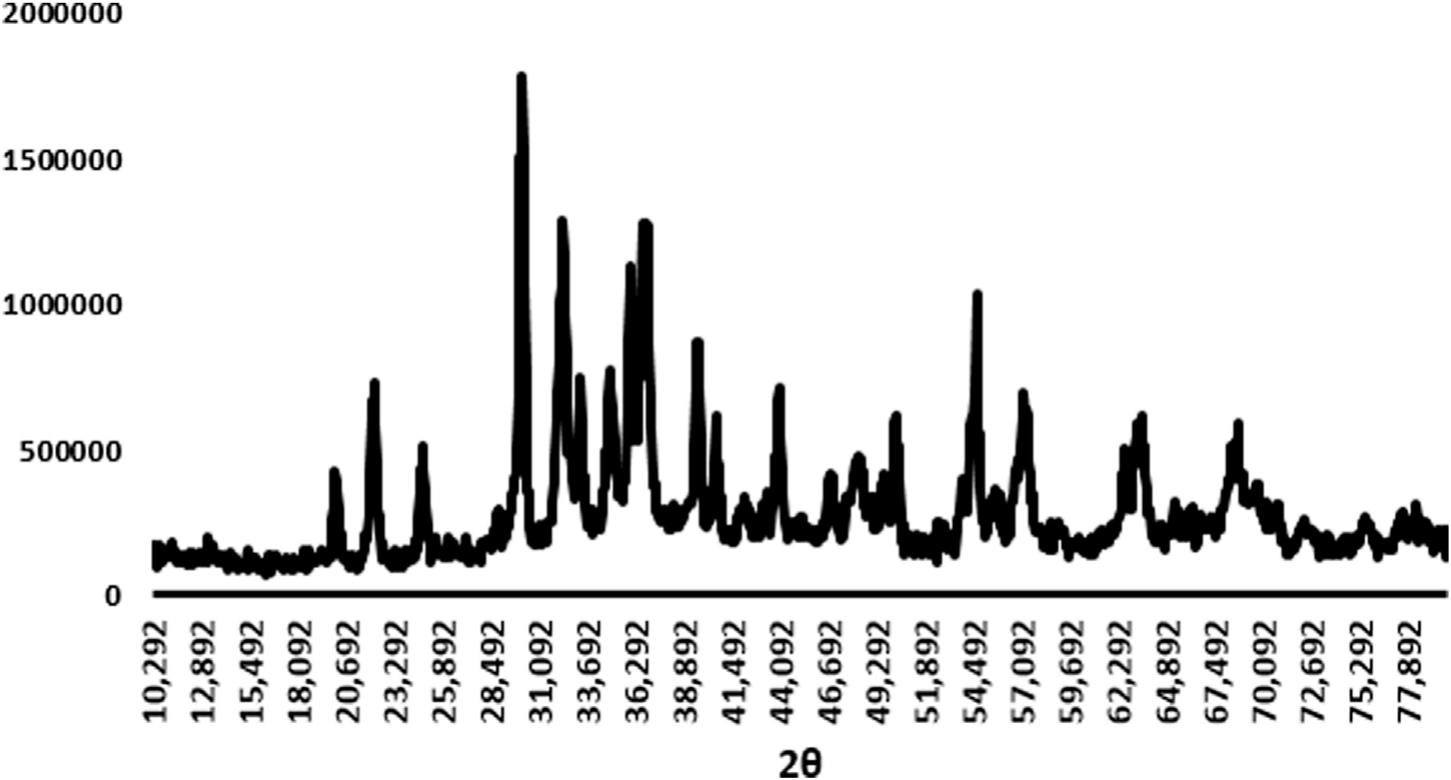
XRD analysis of synthesized AgNPs.

Finally, the XRD patterns of AgNPs showed that the synthesized Ag-Pc has a homogeneous structure (Figure 5). Upon examining the XRD patterns of AgNPs, it is evident that synthesized nanoparticles exhibited consistent Ag and Pc crystal phases. Furthermore, the diffraction pattern of AgNPs showed a broad peak at an angle of approximately 29, 37, and 54° (2θ). This pattern suggests that the intensity of the diffraction peak at around 29° increases in the XRD peak, showing the presence of Ag and Pc. Furthermore, the diffraction peak at 2θ for synthesized AgNPs is broad. Results indicated that the intensity of the diffraction peak at approximately 37° and 54° for the AgNPs is increased, showing the existence of crystal structures of AgNPs.

### 3.2 Hemocompatibility

**FIGURE 6:**
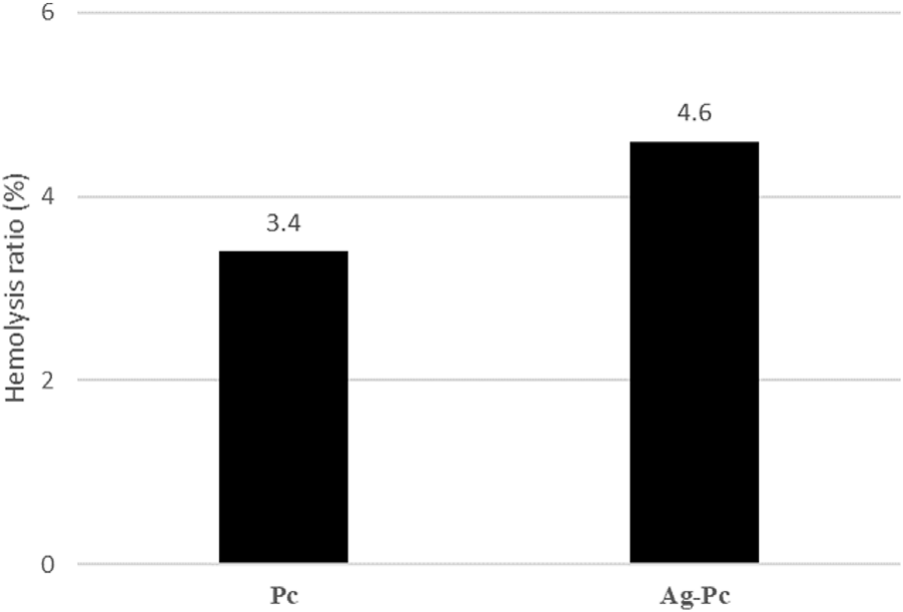
The result of the hemocompatibility assay showed that the Ag-Pc with a 4.6% hemolysis ratio did not induce significant hemolysis.

The Ag-Pc nanoparticles may interact with blood cells that have a negative surface charge, leading to damage to red blood cells (hemolysis) due to their nanometric size. The results of the hemolysis assay (Figure 6) showed that the Ag-Pc did not induce significant hemolysis. Moreover, the incorporation of Ag-Pc reduced the hemolysis.

### 3.3 Toxicity assay

**FIGURE 7:**
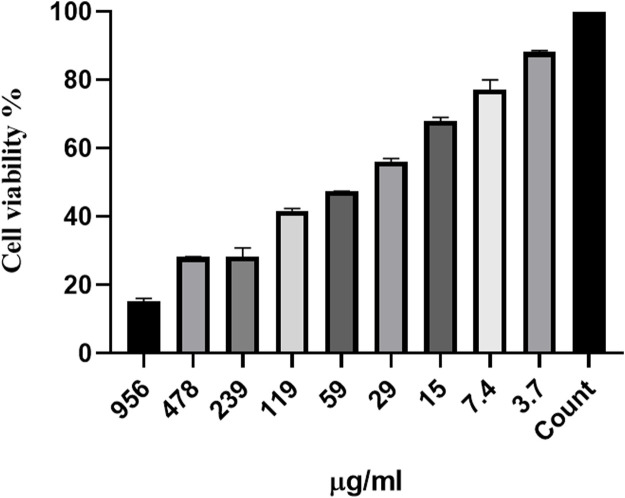
*In vitro* cytotoxicity effect of synthesized Ag-Pc on the murine L929 fibroblasts cell lines. The results of the MTT test showed in the MIC concentration of Ag-Pc for CR *P. aeruginosa* and MRSA, 79.5% of the cells were viable.

Figure 7 shows the effects of different concentrations of Ag-Pc and Pc on the murine L929 fibroblast cell lines. In the MIC concentration of Ag-Pc for CR *P. aeruginosa* and MRSA, 79.5% of the cells were viable. Noteworthy, Pc did not show any effect on cell viability.

### 3.4 The antibacterial effect of synthesized nanoparticles

The MIC and MBC values of the Ag-Pc were assessed and compared to those of the AgNPs and Pc. The MIC of Ag-Pc for MRSA and *P. aeruginosa* was 7.4 μg/mL. On the other hand, Pc did not show antibacterial effects against any of the strains. The MBC of Ag-Pc for the MRSA was 7.4 μg/mL, while it was 14.9 μg/mL for *P. aeruginosa* (Table 2). Our results showed that using Pc to synthesize AgNPs can decrease the MIC and MBC of this nanoparticle against bacteria.

**TABLE 2:** The MICs and MBCs of Ag-PC and Pc.

Isolates	MIC (µg/mL)		MBC (µg/mL)	
	Ag-Pc	AgNPs	Ag-Pc	AgNPs
*MRSA*	7.4	14.9	7.4	14.9
CR *P. aeruginosa*	7.4	14.9	14.9	14.9

### 3.5 Anti-biofilm activity of Ag-Pc (MBIC, MBEC)

**FIGURE 8:**
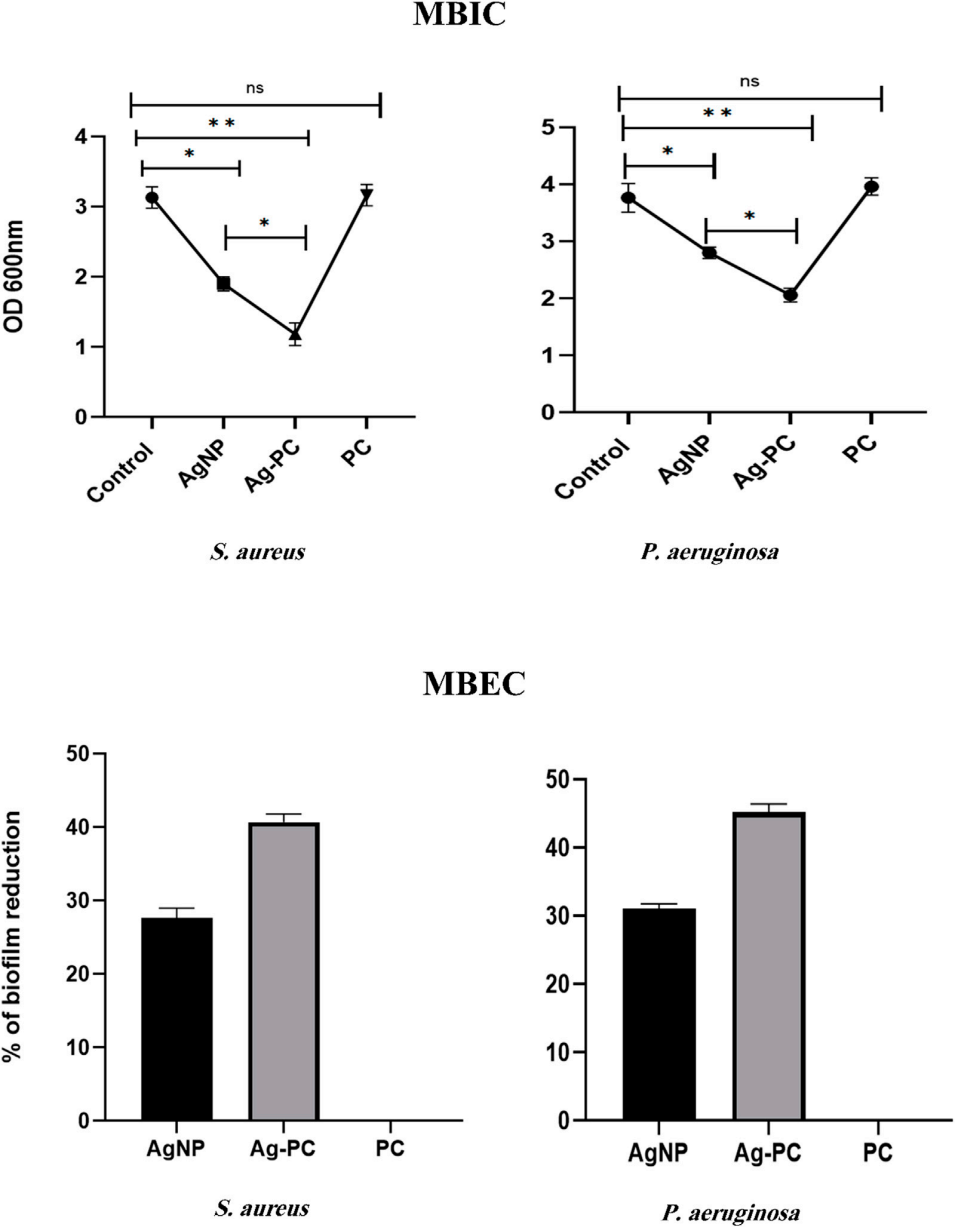
Antibiofilm function of synthesized nanoparticles. MBIC: Ag-Pc showed more inhibitory activity against bacterial biofilms than AgNPs (AgNPs 7.45 μg/mL (1/2 MIC), Ag-Pc 3.7 μg/mL (1/2 MIC) and FC 1 mg/mL). MBEC: Ag-Pc leads to a rapid reduction of biofilm amounting to 49% and 40% detachments in CR *P. aeruginosa* and MRSA, respectively (AgNPs 29.8 μg/mL (2 MIC), Ag-Pc 14.8 μg/mL (2 MIC) and FC 1 mg/mL).)ns: not significant, *: *p* < 0.05, **: *p* < 0.01).

Based on the results, the biofilm production capabilities of the isolates were classified as non-biofilm producers, moderate, and strong. Clinical MRSA and CR *P. aeruginosa* isolates had stronger biofilm formation than ATCC strains. The antibiofilm activity of different concentrations of Ag-Pc was assessed in this stage (Figure 8). Based on the results, it was concluded that 1/2 MIC of Ag-Pc (3.7 μg/mL) suppressed biofilm formation in both of the bacteria more efficiently in comparison to the AgNPs (P< 0.05). Therefore, it was concluded that Ag-Pc can inhibit MRSA and CR *P. aeruginosa* biofilm formation. In another method, spectrophotometric analysis was used to study the susceptibility of established biofilms to different concentrations of components. The findings demonstrated that the MIC of Ag-Pc caused a significant reduction in mature biofilm, with a decrease of 49% in CR *P. aeruginosa* and 40% in MRSA.

### 3.6 The Ag-Pc effect on the biofilm-related genes expression

**FIGURE 9:**
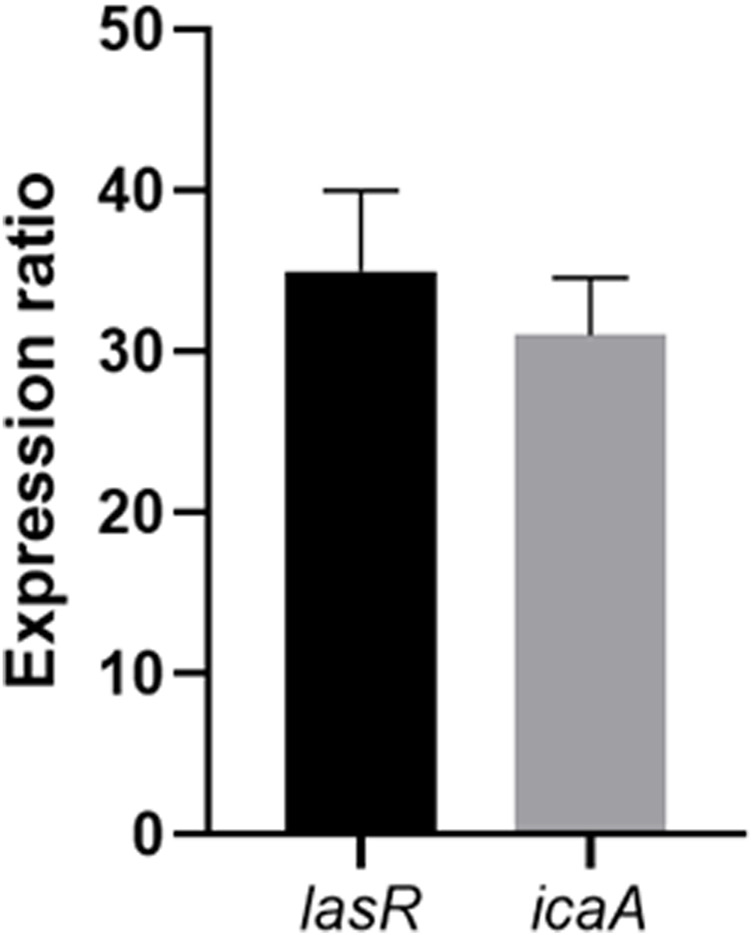
The expression ratio of virulence genes in clinical strains compared to the standard strains.

The expression ratio of virulence genes involved in biofilm formation was assessed in isolated bacteria compared to *S. aureus* ATCC 25923 and *P. aeruginosa* ATCC 27853. The results showed an increase in the virulence genes in clinical strains compared to the standard strains (Figure 9). Following the application of various doses of Ag-Pc to the bacterial biofilms, the RT-PCR analysis was used to measure the relative mRNA expression of genes associated with biofilm formation. The expression levels of the *icaA*, *lasR*, and *algD* genes were measured in MRSA and *CR P. aeruginosa* biofilms in the presence of Ag-Pc. The addition of Ag-Pc did not substantially reduce the expression of all genes relative to the untreated bacteria.

## 4 Discussion

Managing antibiotic-resistant bacteria, particularly their capacity to develop biofilm, is a significant challenge in medicine. These bacteria showed higher antibiotic resistance, and biofilm formation is essential in developing drug resistance (Hosseini et al., 2020). Developing a novel approach for effectively eradicating MDR bacteria is urgently necessitated. Studies have proven that AgNPs have garnered considerable attention due to their efficacy as antibacterial agents, low toxicity, and many applications both *in vitro* and *in vivo* (El-Naggar et al., 2017).

To this end, the first stage of our investigation was a green synthesis and stabilization of AgNPs using Pc as a protective agent, which showed better antibacterial and antibiofilm effects than the AgNPs. The Ag-Pc we synthesized showed antimicrobial properties against CR *P. aeruginosa* and MRSA at a 7.4 μg/mL concentration. In our study, contrary to the results conducted in 2013, Pc alone did not show any antibacterial properties (Leung et al., 2013).

The current study’s findings showed that Ag-Pc significantly decreased the rate of biofilm formation compared to the Pc and AgNPs. Moreover, MBEC detection revealed that Ag-Pc decreased mature biofilm to 49% and 40% in *P. aeruginosa* and *S. aureus*, respectively. Similar to our study, *Terminalia catappa* leaf extract (TCE)-Ag-NPs-3 that was synthesized using the green synthesis method and at a concentration of 7.8 μg/mL significantly inhibits 73.7% biofilm formation in MDR *P. aeruginosa* and 69.56% in MRSA (Ansari et al., 2021). Furthermore, *Goswami* et al. reported that using 15 and 5 μg/mL of AgNPs resulted in an 89% and 20% inhibition of *S. aureus* biofilm formation (Goswami et al., 2015). In this study, the anti-biofilm effect of AgNPs at a high concentration showed a high inhibition percentage; however, the cytotoxicity effect of synthesized AgNPs has not been investigated. This difference in the results of the studies may be related to the dissimilarity of the synthesis compound used. The Ag-Pc synthesized in the current study inhibited the growth of bacteria, destroyed their biofilm community at 5 μg/mL concentration, and did not show a cytotoxicity effect on the murine L929 fibroblasts cell lines at this concentration.

Collectively, as mentioned, the presence of Pc enhanced the antibacterial and antibiofilm function of AgNPs; actually, the improvement of antibacterial activity by AgNPs may be related to the protective role of Pc. The C-Pc contains functional groups, including amino, carboxyl, and mercapto groups, which can serve as protective agents for nanoparticles when creating AgNPs. The silver atom has a pronounced attraction towards these functional groups, resulting in the stable synthesis of nanomaterials (Guo and Irudayaraj, 2011; Wei et al., 2018). Extracellular reduction of Ag + ions occurs via reductase enzymes and electron shuttle quinones (Durán et al., 2005). Within the cells, Ag + ions undergo reduction by utilizing electrons generated by the organisms. This process can prevent their destruction, particularly in the presence of enzymes like NADH-dependent reductases (Anil Kumar et al., 2007). C-Pc is a protective agent that extracellularly reduces Ag + ions (Wei et al., 2018). Ag + can induce oxidative stress and damage DNA in bacterial cells (Xu et al., 2021). Therefore, Pc can improve the antibacterial and antibiofilm function of AgNPs; however, data in this field is limited, and more confirmatory studies are needed.

Finally, our findings indicated that treatment of bacterial biofilms in sub-MIC concentrations of Ag-Pc and Pc had no significant effect on the biofilm-related gene expression. Similar to our findings, the results of the recently published study also indicated that the expression of icaA and icaD genes did not significantly reduce the presence of curcumin, silver, and zinc nanoparticles compared to the control (Gheidar et al., 2018). Nevertheless, the study’s results indicated a remarkable reduction in the *icaR* gene expression at a dose of 5 μg/mL, compared to a concentration of 3 μg/mL for the *S. epidermidis* strain. The authors hypothesized that these disparities may be attributed to the suppressive impact of the gene at a dose of 3 μg/mL and the absence of biofilm development at 5 μg/mL (Swolana et al., 2022). In addition, *Wang* et al. discovered that a titanium surface modified with AgNPs can control the expression levels of genes associated with biofilm formation (*icaR* and *icaA* and *fnbA* and *fnbB* for *S. epidermidis* and *S. aureus,* respectively). This regulation helps prevent bacterial adherence and the formation of biofilms (Wang et al., 2016). Therefore, there are contradictory results on the effect of AgNPs on biofilm-related gene expression. Notably, different genes and nanoparticle types and concentrations were used in the mentioned studies. To this end, more confirmatory studies are needed to understand better the interaction between AgNPs and the gene expression associated with the bacteria attachment and formation of the biofilm community.

## 5 Conclusion

This study employed the green synthesis approach to produce the Ag-Pc molecule, which exhibited potent antibacterial and antibiofilm properties against MRSA and CR *P. aeruginosa,* even at low concentrations. These findings indicate the potential utility of Ag-Pc in treating infections caused by drug-resistant organisms that generate biofilms. The primary benefit of utilizing plant extracts for the green synthesis of AgNPs is the promotion of environmental and human health preservation by reducing dangerous chemical usage. Furthermore, this study also demonstrated the potential use of Ag-Pc as a significant medicinal and clinical alternative. Nevertheless, further extensive research is required to determine the therapeutic feasibility of utilizing this category of substances. Further investigation into the antibacterial processes of Ag-Pc will enhance our ability to eradicate bacterial infections in clinical environments efficiently.

## Data Availability

The original contributions presented in the study are included in the article/Supplementary Material, further inquiries can be directed to the corresponding authors.
