# Correction: Antibacterial and antibiofilm activity of silver nanoparticles stabilized with C-phycocyanin against drug-resistant *Pseudomonas aeruginosa* and *Staphylococcus aureus*

**DOI:** 10.3389/fbioe.2025.1714279

**Published:** 2025-10-28

**Authors:** Zahra Chegini, Aref Shariati, Mohammad Yousef Alikhani, Maliheh Safaiee, Shahin Rajaeih, Mohammadreza Arabestani, Mehdi Azizi

**Affiliations:** ^1^ Department of Microbiology, School of Medicine, Hamadan University of Medical Sciences, Hamadan, Iran; ^2^ Infectious Diseases Research Center (IDRC), Arak University of medical sciences, Arak, Iran; ^3^ Department of Organic Chemistry, Faculty of Chemistry and Petroleum Sciences, Bu-Ali Sina University, Hamadan, Iran; ^4^ ENT and Head and Neck Research Center and Department, The Five Senses Health Institute, Iran University of Medical Sciences, Tehran, Iran; ^5^ Infectious Diseases Research Centre, Hamadan University of Medical Sciences, Hamadan, Iran; ^6^ Department of Tissue Engineering and Regenerative Medicine, Hamadan University of Medical Sciences, Hamadan, Iran

**Keywords:** silver nanoparticles, C-phycocyanin, biofilms, antibiotics-resistant, new treatment

In the published article, there was an error in the **Funding** statement. The grant number, “14020503305,” is incorrect in the funding statement. The grant number should be “140205033505.” The correct **Funding** statement appears below.

“The author(s) declare financial support was received for this article’s research, authorship, and/or publication. This research was supported by grant no. 140205033505 from the Hamadan University of Medical Sciences”.

The original article has been updated.

