# Supplementary figures and images for: Antibacterial and antibiofilm activity of silver nanoparticles stabilized with C-phycocyanin against drug-resistant Pseudomonas aeruginosa and Staphylococcus aureus
Source: Front Bioeng Biotechnol. 2024 Oct 23;12:1455385. doi: 10.3389/fbioe.2024.1455385 (PMC11544008; doi:10.3389/fbioe.2024.1455385)

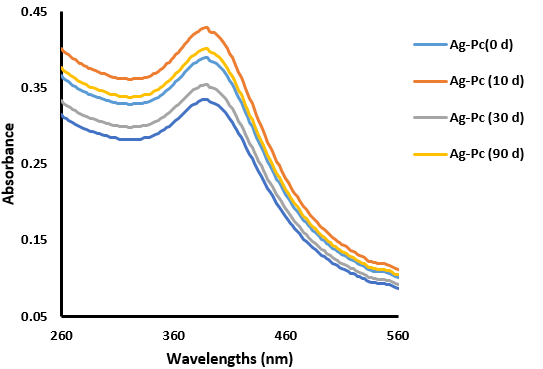


Figure S1. UV-vis spectra of synthesized nanoparticles after three months.

Supplement: Supplementary file 1 [file DataSheet1.docx]
